# The Therapeutic Potential of Mesenchymal Stem Cells in Post-Stroke Depression

**DOI:** 10.3390/ijms27114796

**Published:** 2026-05-26

**Authors:** Manru Fan, Que Deng, Zhimin Li, Guibin Wang, Ming Lu

**Affiliations:** 921 Hospital of Joint Logistics Support Force People’s Liberation Army of China (the Second Affiliated Hospital of Hunan Normal University), Changsha 410003, China; fanmanru@163.com (M.F.); dengque3s@163.com (Q.D.); bjyx97105@163.com (Z.L.)

**Keywords:** post-stroke depression, mesenchymal stem cells, mechanisms, review

## Abstract

Post-stroke depression (PSD) is the most prevalent neuropsychological disorder among stroke survivors, affecting over 30% of patients. It significantly impairs patients’ quality of life and imposes a substantial burden on individuals, families, and society. Currently, the primary treatment for PSD focuses on conventional antidepressant therapies, with a lack of innovative approaches. Therefore, there is an urgent need to develop novel targeted therapies for PSD. This review synthesizes PSD pathogenesis as a multi-system network disorder involving monoamine deficits, neuroinflammation, HPA axis dysfunction, and neurotrophic imbalance. Within this framework, mesenchymal stem cells (MSCs) transplantation, as an emerging therapeutic strategy, may exert beneficial effects through anti-inflammatory, neuroprotective mechanisms, and the provision of neurotrophic factors. This review provides a preclinical framework that highlights the potential of MSC-based strategies, while emphasizing the need for further validation in PSD-specific models before clinical translation.

## 1. Introduction

Stroke is a very common and harmful cardiovascular disease that often leads to limb movement function and neuropsychological disorders. According to the 2019 Global Burden of Disease Study [1], stroke remains the second leading cause of death and the third leading cause of combined mortality and disability worldwide. The incidence and prevalence of stroke-related complications are increasing globally [2]. Post-stroke depression (PSD), characterized by sustained emotional decline and decreased interest [3], is the most prevalent neuropsychological disorder among stroke survivors, affecting over 30% of this population [4,5]. Currently, it is becoming increasingly accepted that PSD has a strong association with increased mortality, reduced quality of life, poorer rehabilitation outcomes, and worse functional recovery in stroke survivors [4]. Broadly, the treatment strategies for PSD encompass pharmacological and non-pharmacologic approaches. However, previous research has indicated that conventional pharmacotherapy, particularly first-line antidepressants, demonstrates limited efficacy in addressing mood and cognitive impairments associated with stroke [6]. Furthermore, standard pharmacological regimens often require extended durations (6 months to 1 year) and may be constrained by their negative side effects, poor compliance, and delayed therapeutic response [7,8]. Non-pharmacological alternatives, while available, are frequently costly, necessitate specialized expertise, but yield limited efficacy [9,10]. Consequently, current primary interventions for PSD remain predominantly focused on symptomatic depression management, with a distinct lack of novel, targeted therapeutic options. Therefore, it is urgent to develop new targeted treatments specifically for PSD.

Albeit in their infancy, cell-based therapy represents a promising approach for treating neurodegenerative disease [11,12]. Cell source choices remain critical for such therapies. While induced pluripotent stem cells (iPSCs) have been considered a potential source since 2006, their clinical translation remains constrained by cost-effectiveness concerns and adverse events, notably tumorigenesis [13]. Among the various sources of stem cells, mesenchymal stem cells (MSCs) are one of the ideal cell types for clinical applications due to their easy of isolation, culture expansion in vitro, stable phenotype maintenance in vitro and the ability to home to injury sites upon administration in vivo [14]. MSCs, originally identified as adherent fibroblast-like cells capable of mesodermal differentiation (e.g., into osteocytes, chondrocytes, and adipocytes), also exhibit pluripotent differentiation potential across embryonic layers (ectoderm and endoderm) and possess robust self-renewal capacity [15]. Although being initially identified from bone marrow, MSCs have subsequently been derived from diverse tissues including adipose tissue, pancreas, liver, skeletal muscle, dermis, synovial membrane, trabecular bone [16], umbilical cord blood [17], lung tissue [18], olfactory mucosa [19], and dental pulp and periodontal ligament [20]. They exhibit therapeutic effects through various mechanisms and have immunomodulatory functions, which make them widely used in medical research [21]. Central nervous system (CNS) disorders, such as Parkinson’s disease (PD), traumatic brain injury (TBI), and stroke, are considered promising therapeutic targets for MSC transplantation, with several clinical trials already undertaken [19,22]. However, investigation into their application for PSD remains notably limited. While several recent reviews have surveyed the landscape of stem cell therapy for major depressive disorder or neurological conditions, none has specifically addressed the distinct neuropsychiatric context of PSD or examined MSC-based interventions within a disease-specific framework [23,24,25]. Given this scarcity of PSD-specific studies, this review draws upon mechanistic insights from adjacent fields, including general depression, stroke without mood comorbidities, and neuroinflammatory conditions, to construct a conceptual basis for MSCs as a promising therapeutic strategy for PSD.

PSD significantly impairs patients’ quality of life, interpersonal skills, and work capacity, imposing a substantial burden on society. Consequently, early intervention and timely treatment are paramount. MSCs transplantation, as an emerging therapeutic approach, holds the potential to ameliorate mood and mental health by inhibiting neuroinflammation, exerting neuroprotective effects, and providing neurotrophic factors (NTFs). This review aims to explore the therapeutic potential of MSC transplantation for PSD, analyze its underlying mechanisms of action, and establish a preclinical foundation to guide future research in PSD-specific models.

## 2. The Pathogenesis of PSD

**Figure 1:**
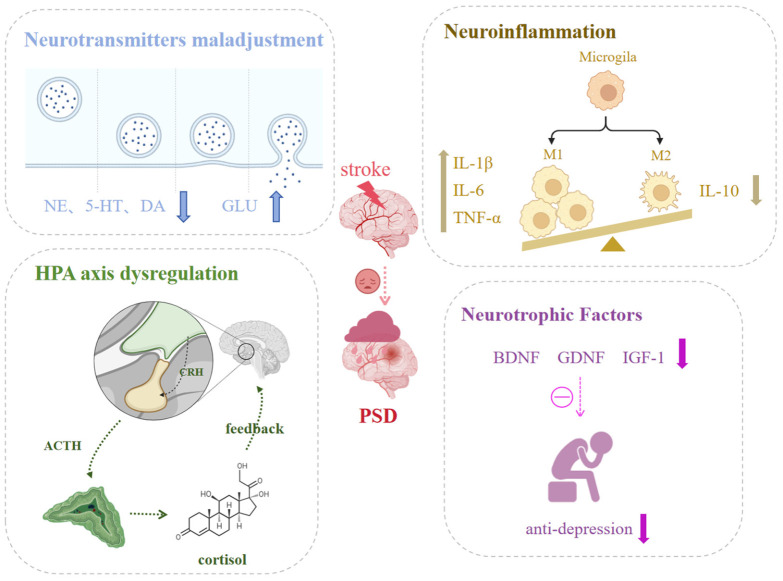
The pathogenesis of PSD includes four aspects: neurotransmitters maladjustment, neuroinflammation, HPA axis dysregulation, and NTFs. PSD, post-stroke depression; NE, norepinephrine; 5-HT, 5-hydroxytryptamine; DA, dopamine; GLU, glutamate; IL-1β, interleukin-1β; IL-6, interleukin-6; TNF-α, tumor necrosis factor-α; IL-10, interleukin-10; HPA, hypothalamic–pituitary–adrenal; CRH, corticotropin-releasing hormone; ACTH, adrenal cortical hormone; BDNF, brain-derived neurotrophic factor; GDNF, glial cell line-derived neurotrophic factor; IGF-1, insulin-like growth factor-1; NTFs, neurotrophic factors.

PSD involves both neurological and psychiatric components, resulting in notably intricate etiology and pathogenesis [26]. Existing evidence supports that the joint involvement of alterations in neurotransmitters, inflammatory responses, neuroendocrine activation, and NTFs dysregulation likely contribute to the disease process [3,27], with these mechanisms exhibiting complex crosstalk. The following sections will systematically elucidate the pathogenesis of PSD, focusing on four key dimensions: neurotransmitters maladjustment, neuroinflammation, neuroendocrine, and NTFs (Figure 1).

### 2.1. Neurotransmitters Maladjustment

At present, the main theories about the neurotransmitters of PSD include the monoamine hypothesis and the glutamate-mediated excitotoxicity hypothesis [3]. These mechanisms are distinguished by their mode of action: monoamine dysfunction directly precipitates depressive symptoms, whereas glutamatergic excitotoxicity indirectly damages the neural circuitry underlying emotional regulation.

Substantial neurophysiological evidence identifies monoamine neurotransmitters and genes as among the most critically implicated pathogenesis in PSD. Monoamine neurotransmitters mainly include norepinephrine (NE), 5-hydroxytryptamine (5-HT), and dopamine (DA), which transmit messages between nerve cells or neurons and effector cells, integrating the overall coordination of body functions. Critically, monoamine depletion shows a direct causal relationship with depressive phenotypes, unlike mechanisms operating through indirect neuronal damage. Clinical studies consistently demonstrate reduced monoamine levels in depressed patients versus controls, establishing these neurotransmitters as core biological markers of depression [28]. A key insight is the region-specific vulnerability of monoamine systems following stroke. NE and 5-HT exhibit greater depletion susceptibility in the left hemisphere, correlating with higher depression incidence after left-sided lesions-particularly in the frontal cortex and basal ganglia [26]. This anatomical selectivity suggests that neurochemical consequences are as critical to PSD as lesion location itself. This left-hemispheric vulnerability likely reflects a mechanism unique to PSD that is absent in primary depression. Ascending monoaminergic fibers from the brainstem pass through the medial forebrain bundle to innervate cortical and subcortical targets. Ischemic lesions that physically interrupt these fiber tracts directly compromise neurotransmitter delivery to limbic and prefrontal regions, establishing a structural basis for monoamine depletion that is fundamentally distinct from the functional dysregulation seen in non-vascular depression. Additionally, 5-HT interacts with 14 gastrointestinal receptor subtypes, introducing peripheral–central regulation via the gut–brain axis (GBA) [29]. DA, synthesized from tyrosine (Tyr) with tetrahydrobiopterin (BH4) as cofactor, further contributes to emotional regulation, with animal studies confirming reduced monoamine concentrations in frontal cortex and hippocampus (HIP) of PSD-model rats [3,30]. Collectively, these findings position monoamine dysfunction as a direct mechanistic driver of PSD, not merely a disease correlate.

While the monoamine hypothesis addresses neurochemical deficits, the glutamate-mediated excitotoxicity hypothesis focuses on neuronal injury as a complementary mechanism. Many studies have reported higher levels of glutamate and its metabolites in both the blood and cerebrospinal fluid of patients with PSD, especially in the frontal cortex [31,32]. Acute cerebrovascular events trigger a surge in glutamate that spreads beyond the infarct core, where excessive receptor overstimulation initiates excitotoxic cascades involving cellular edema, apoptosis, and neuronal death. This cascade underpins neurofunctional impairment and the subsequent manifestation of psychiatric sequelae such as depression, anxiety, and dementia. Unlike primary depression, where glutamate fluctuations are modest and chronic, the excitotoxic process in PSD is triggered by an acute ischemic event and may follow a biphasic course. The initial surge of glutamate within hours of stroke is followed by a sustained phase of milder but persistent excitotoxicity driven by microglial activation and pro-inflammatory mediators. This transition from acute fulminant to inflammation-sustained glutamate dysregulation is a distinctive feature of PSD and may contribute to its treatment resistance, although direct validation of this temporal model remains limited.

Crucially, glutamate excitotoxicity may function synergistically with monoamine dysfunction. Emerging evidence suggests glutamate directly activates the hypothalamic–pituitary–adrenal (HPA) axis, affecting corticotropin-releasing hormone (CRH), adrenal cortical hormone (ACTH), and glucocorticoid (GC) secretion [33]. This provides a mechanistic bridge linking excitotoxic injury to neuroendocrine dysregulation, which in turn exacerbates monoamine deficiency. Furthermore, as discussed in Section 2.4, excessive N-methyl-D-aspartate (NMDA) receptor activation may also disrupt the proBrain-derived neurotrophic factor (BDNF)/mature BDNF (mBDNF) equilibrium, establishing an additional link between excitotoxicity and neurotrophic dysregulation. Thus, these hypotheses likely describe different temporal stages of PSD: excitotoxicity initiates neuronal damage and HPA axis dysregulation, while monoamine depletion maintains the depressive phenotype.

### 2.2. Neuroinflammation

Neuroinflammation is considered as the primary cause of secondary injury following a stroke and is postulated to play a crucial role in the onset and progression of PSD [34]. Stroke-induced immune activation triggers a cascade of inflammatory events that disrupt monoaminergic and neuroendocrine function, ultimately precipitating depressive symptoms [35,36]. This section examines the cellular mediators, molecular effectors, and systemic consequences of neuroinflammation in PSD pathogenesis.

Microglia serve as the principal cellular mediators of neuroinflammation in PSD. Among the most diverse cells in the CNS, microglia exhibit heterogeneity across developmental stages, colonization sites, and disease states. Following CNS injury, microglia detect abnormal signals through surface receptors and undergo activation, characterized by enhanced phagocytosis and morphological changes [36,37]. Critically, microglial function is dynamically regulated by the duration and nature of the insult. In the early stages of injury, microglia predominantly release anti-inflammatory factors to repair damaged neurons. However, persistent insults trigger a phenotypic shift toward a pro-inflammatory state, characterized by secretion of factors that amplify neuroinflammation and exacerbate neuronal damage. This activation is traditionally dichotomized into neurotoxic M1-phenotypes (expressing tumor necrosis factor-α [TNF-α] interleukin-1β [IL-1β], interleukin-6 [IL-6], inducible nitric oxide synthase [iNOS], C-C motif chemokine ligand 2 [CCL2]), and neuroprotective M2-phenotypes (releasing Ym1, Arginase 1, Interleukin-10 [IL-10], Interleukin-10 [IL-4], transforming growth factor-β [TGF-β]) [38,39]. The clinical relevance of this mechanism is supported by evidence that chronic stress-induced depression-like behavior in ischemic stroke rats is associated with microglial activation in the ipsilateral HIP. The clinical relevance of this mechanism is supported by evidence that chronic stress-induced depression-like behavior in ischemic stroke rats is associated with microglial activation in the ipsilateral hippocampus [40]. Furthermore, therapeutic regulation of microglia through multiple intracellular pathway has been shown to inhibit neuroinflammation and improve PSD-like behaviors in rats [41]. Importantly, the neuroinflammatory response in PSD differs fundamentally from that observed in conventional rodent stroke models that predominantly affect gray matter, where injury typically initiates a moderate, self-limiting inflammatory response [42]. In contrast, the human brain is rich in white matter, and damaged myelin sheaths provide a highly pro-inflammatory target that drives severe, destructive, and prolonged inflammation. A meta-analysis confirmed a significant association between white matter hyperintensities, particularly periventricular ones, and chronic-phase PSD [43]. Myelin debris is cleared inefficiently in the CNS, perpetuating microglial activation long after the initial insult and sustaining a chronic pro-inflammatory state that contributes to the treatment resistance of PSD. Future preclinical studies should therefore prioritize animal models that incorporate white matter injury and chronic cerebral hypoperfusion, combined with validated stress protocols, to more faithfully recapitulate the human PSD pathology and enable the identification of effective anti-inflammatory interventions.

Soluble inflammatory cytokines function as key molecular effectors linking neuroinflammation to PSD. Previous studies found that the increased levels of pro-inflammatory cytokines, including IL-1β, IL-6, TNF-α, and the decreased levels of anti-inflammatory cytokines, such as IL-10, are strongly associated with PSD [44,45,46]. These cytokines directly impact brain regions critical for mood regulation, potentially precipitating depressive symptoms. IL-1β plays a dual role in PSD pathogenesis, driving post-stroke neuroinflammation and neurodegeneration while concurrently mediating stress-induced depressive-like behaviors [47]. Clinical investigations have highlighted elevated serum IL-1β levels in individuals with PSD compared to their non-PSD counterparts [48]. IL-6 represents another critical mediator with prognostic value. Within the CNS, IL-6 is synthesized by an array of cells including neurons, astrocytes, microglial cells, and endothelial cells, exerting crucial pro-inflammatory effects in PSD pathogenesis. Elevated IL-6 during acute stroke independently predicts depression at two weeks and one-year post-stroke [49], and correlates with physical symptom severity [36]. TNF-α contributes to PSD through effects on synaptic plasticity. Studies have demonstrated increased serum TNF-α levels in PSD patients that positively correlate with depression severity [50]. Conversely, anti-inflammatory cytokines exert protective effects. IL-10 serves as a protective factor against PSD development, with its levels correlating positively with stroke outcomes and quality of life [51]. Lower levels of IL-10 post-stroke have been linked to an increased risk of PSD, underscoring its neuroprotective role [52]. Beyond microglia and cytokines, astrogliosis also contributes to the post-stroke inflammatory milieu in ways relevant to PSD [53]. Reactive astrocytes have been implicated in limiting the spread of tissue damage through anti-inflammatory responses, clearing extravasated red blood cells and debris via erythrophagocytosis, and removing excess fluid in peri-lesional vasogenic edema. Vasogenic edema, a hallmark of severe destructive inflammatory disease with a prolonged course, is particularly prominent in white matter-rich regions. The failure or exhaustion of these astrocytic functions may therefore exacerbate and prolong post-stroke neuroinflammation, further entrenching the pathological circuits that underlie depressive symptoms.

Collectively, dysregulation in the pro-inflammatory and anti-inflammatory cytokine balance constitutes a pivotal mechanism in PSD’s chronic trajectory. This neuroinflammatory process is amplified in the white matter-rich human brain, where myelin debris sustains microglial activation and reactive astrocyte dysfunction fails to resolve vasogenic edema, perpetuating a prolonged inflammatory state that distinguishes PSD from non-vascular depression. This framework positions neuroinflammation not merely as a stroke consequence, but as a central pathogenic driver and potential therapeutic target in PSD.

### 2.3. Hypothalamic–Pituitary–Adrenal Axis Dysregulation

The HPA axis constitutes the primary neuroendocrine system regulating affective states, and its hyperactivation constitutes a core pathogenic mechanism in PSD. Importantly, the HPA axis interacts bidirectionally with both the monoaminergic system and neuroinflammation, forming a complex network that perpetuates PSD pathology [3,33].

Under physiological conditions, HPA axis function follows a classic cascade. The hypothalamic paraventricular nucleus releases CRH, stimulating pituitary ACTH secretion, which in turn triggers GCs release from the adrenal cortex. GCs then exert negative feedback via hippocampal and hypothalamic pathways to maintain homeostasis [54,55]. n PSD, this regulatory mechanism is disrupted, resulting in HPA axis hyperactivity [56]. Hypercortisolemia is consistently reported in depressed individuals, accompanied by peripheral 5-HT downregulation [57]. PSD patients specifically show elevated afternoon cortisol with disrupted diurnal rhythm [58], insensitivity in the dexamethasone suppression test (DST) [59], and animal models demonstrate increased corticosterone reversible by antidepressants [60]. Critically, hypercortisolism predicts later PSD development, suggesting a causal role [59]. While HPA axis hyperactivity is a well-recognized feature of major depression, in PSD it is triggered and sustained by stroke-specific pathological events. The acute ischemic insult directly activates the HPA axis through multiple converging pathways. Excessive glutamate release stimulates CRH secretion from the hypothalamus, pro-inflammatory cytokines such as IL-1β and IL-6 potently activate the axis at both hypothalamic and adrenal levels, and damage to the hippocampus, a structure highly vulnerable to ischemia, impairs the glucocorticoid-mediated negative feedback that normally restrains HPA activity.

The HPA axis interacts synergistically with serotonergic and inflammatory systems. It influences serotonergic function by modulating 5-HT and serotonin transporter gene-linked promoter region (5-HTTLPR) binding, thereby altering cortisol dynamics [61]. This bidirectional relationship links HPA dysfunction directly to the monoamine deficits discussed in Section 2.1. Concurrently, HPA dysregulation drives neuroinflammation. Dysfunction stimulates pro-inflammatory cytokine expression, which in turn further activates the HPA axis and elevates corticosterone, creating a self-perpetuating cycle. Chronic inflammatory stress exacerbates this imbalance, amplifying neurological damage [56]. This reciprocal activation directly engages the neuroinflammatory mechanisms detailed in Section 2.2.

Collectively, the HPA axis serves as a central hub integrating the neuroendocrine, monoaminergic, and inflammatory dimensions of PSD. Its hyperactivity not only directly contributes to depressive symptoms but also amplifies serotonergic dysfunction and sustains neuroinflammation. In PSD, this central hub function takes on particular significance because the initial ischemic injury simultaneously damages the hippocampal negative-feedback apparatus and provides sustained inflammatory drive, locking the HPA axis into a chronically activated state that is more resistant to resolution than in non-vascular depression. Targeting HPA normalization may therefore represent a multi-target therapeutic strategy, though effective intervention will likely require concurrent engagement of the upstream ischemic and inflammatory drivers that perpetuate its dysfunction.

### 2.4. Neurotrophic Factors

NTFs constitute a class of peptides or small proteins in the CNS that play a crucial role in neuronal growth, differentiation, and survival. These molecules serve as essential regulators of brain development, homeostasis, and plasticity.

BDNF is the most extensively studied NTFs in depression research. Primarily expressed in the HIP and cerebral cortex [62], BDNF participates in both physiological and pathological processes underlying depression and ischemic stroke [63,64]. The causal relationship between BDNF and depressive phenotypes is well-established: promoting BDNF expression alleviates depressive symptoms, while BDNF gene deletion exacerbates them [65,66]. This positions BDNF as both a pivotal determinant in depression etiology and a prime therapeutic target. Critically, BDNF exists in two functionally opposed isoforms. ProBDNF is implicated in inflammatory and apoptotic pathways, whereas mBDNF binds tyrosine kinase receptor (TrkB) to mediate cellular proliferation, survival, synaptogenesis, and memory consolidation [67]. Critically, the proBDNF/mBDNF equilibrium plays a decisive role in PSD development [68]. Rodent PSD models exhibit significantly elevated serum proBDNF levels with reduced mBDNF/proBDNF ratios, and this imbalance correlates with diminished hippocampal neurofunction [69]. This proBDNF/mBDNF imbalance in PSD is driven by the unique confluence of post-stroke pathology. Ischemia-induced excitotoxicity and sustained neuroinflammation promote proBDNF cleavage toward its pro-apoptotic isoform while simultaneously suppressing mBDNF-TrkB signaling, a dual disruption more pronounced than in non-vascular depression.

Other NTFs also contribute to PSD pathogenesis, though their roles are less defined. Glial cell line-derived neurotrophic factor (GDNF), widely distributed in the hypothalamus, shows reduced levels in depressive states that are elevated by antidepressant therapies [70]. Clinical evidence identifies GDNF and its mRNA as potential biomarkers for differentiating major depressive disorder from PSD [71]. It further suggests that GDNF may act on neurotransmitters and thus participate in the development of PSD. Insulin-like growth factor-1 (IGF-1) has received much attention for its influence on recovery after stroke [72]. Ketamine, an NMDA receptor antagonist with rapid antidepressant properties, induces sustained IGF-1 release in the male murine prefrontal cortex. Low serum IGF-1 levels on admission may predict PSD development, and carriers of the T allele at the rs9282715 locus of the IGF-1R gene may show heightened susceptibility [46].

PSD pathogenesis arises from the complex interplay of multiple interdependent systems rather than a single pathological mechanism. Monoaminergic dysfunction, particularly region-specific depletion of NE, 5-HT, and DA, constitutes the direct neurochemical substrate of depressive symptoms. This deficit, however, is closely intertwined with neuroinflammation and HPA axis dysregulation. Microglial activation and pro-inflammatory cytokines disrupt monoamine metabolism, activate the HPA axis, and shift BDNF signaling toward pro-apoptotic pathways. Concurrently, HPA axis hyperactivation elevates GCs, which exacerbate serotonergic deficits, sustain neuroinflammation, and impair hippocampal neuroplasticity. Adding to this complexity, glutamate excitotoxicity, which is discussed in Section 2.1 as a complementary mechanism of neuronal injury in PSD, directly intersects with BDNF signaling. Excessive glutamate release and subsequent NMDA receptor overstimulation promote proBDNF cleavage toward its pro-apoptotic isoform while suppressing mBDNF-TrkB-mediated survival pathways, thereby linking excitotoxic injury to neurotrophic imbalance. These interconnected pathological processes converge on BDNF dysregulation as a key molecular node, through which inflammatory, endocrine, monoaminergic, and excitotoxic disturbances translate into impaired neuronal survival and plasticity. The reduction of GDNF and IGF-1 in PSD further illustrates how stroke-induced neuroinflammation and HPA axis dysfunction converge to suppress multiple neurotrophic pathways, compounding the deficit beyond BDNF alone. Therefore, PSD is best conceptualized as a multi-system network disorder in which monoamine deficits, neuroinflammation, HPA axis dysfunction, and glutamate-mediated excitotoxicity interact bidirectionally, with BDNF serving as a critical downstream mediator of their cumulative effects.

## 3. Treatments

**Figure 2:**
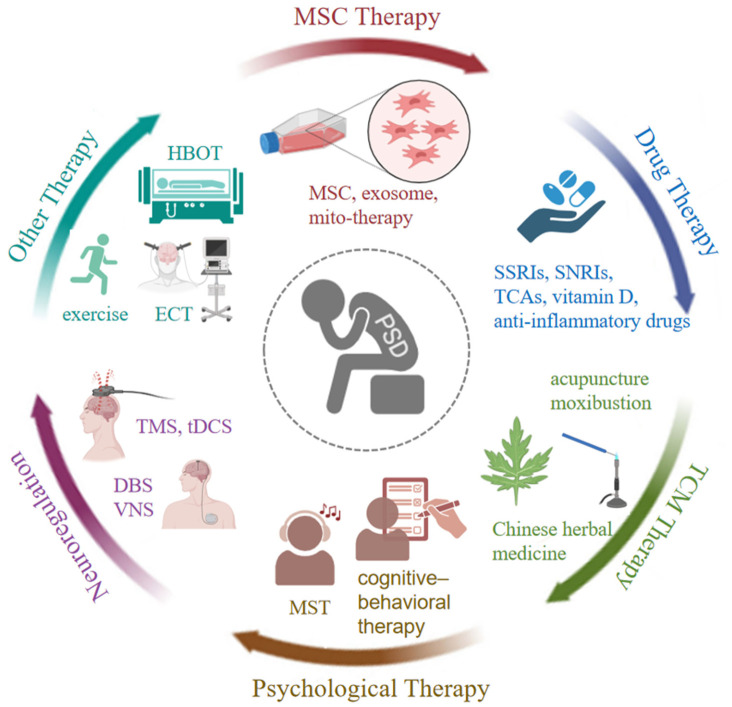
The main treatments of PSD, including six aspects: MSC therapy, drug therapy, TCM therapy, psychotherapy therapy, neuromodulation, and other therapy. PSD, post-stroke depression; MSC, mesenchymal stem cell; SSRIs, serotonin-reuptake inhibitors; SNRIs, serotonin–norepinephrine reuptake inhibitors; TCAs, tricyclic antidepressants; TCM, traditional Chinese medicine; MST, music support therapy; TMS, transcranial magnetic stimulation; tDCS, transcranial direct current stimulation; DBS, deep brain stimulation; VNS, vagus nerve stimulation; HBOT, hyperbaric oxygen treatment; ECT, electroconvulsive therapy.

The multifactorial pathophysiology of PSD poses a formidable challenge to its treatments. Currently, the treatments primarily involve pharmacological therapy, while non-pharmacological therapy encompasses traditional Chinese medicine (TCM), psychotherapy, neuromodulation, and other treatments (Figure 2). The major therapeutic approaches for PSD and their proposed mechanisms of action are summarized in Table 1.

### 3.1. Drug Therapy

Drugs mainly include selective serotonin-reuptake inhibitors (SSRIs), serotonin–norepinephrine reuptake inhibitors (SNRIs), tricyclic antidepressants (TCAs), anti-inflammatory drugs, vitamin D, etc., which have been confirmed to have better efficacy by clinical studies [26,73]. Building upon the multi-system network model of PSD pathogenesis discussed in Section 2, pharmacological interventions target various nodes within this network. SSRIs, SNRIs, and TCAs directly address monoaminergic dysfunction, while anti-inflammatory agents and vitamin D target upstream inflammatory and neurotrophic pathways.

#### 3.1.1. Antidepressant Drugs

SSRIs, SNRIs, and TCAs represent the mainstay of pharmacological treatment for PSD. SSRIs remain the first-line treatment due to their established efficacy in improving depressive and anxiety symptoms, cognitive function, and daily living abilities, while also alleviating associated adverse effects such as headache and insomnia [55,74,75]. Additionally, post-stroke use of SSRIs also prevents the development of PSD [76]. Nevertheless, one study found no improvement in depressive symptoms with SSRI treatment [77]. In addition to the negative results mentioned above, the long-term benefits of SSRIs remain inconclusive. SSRIs present potential risks of bleeding and fractures [78], and may disrupt platelet function, thereby increasing hematoma volume and the risk of recurrent hemorrhagic stroke [79]. SNRIs are another class of antidepressants introduced in 1993 and are effective in treating depression and improving emotional awareness in PSD patients [73]. Their mechanism of action directly addresses the monoamine deficits discussed in Section 2.1: by blocking the reuptake of both serotonin and NE, they increase neurotransmitter concentrations in the synaptic cleft, thereby stimulating descending inhibitory pathways in the spinal cord. However, despite their documented efficacy, SNRIs also face limitations in specific populations, such as pregnant or breastfeeding women, due to potential fetal exposure. TCAs similarly target both neurotransmitters. Meta-analyses have demonstrated their superior efficacy compared to placebo [80] and comparable effectiveness to SSRIs and SNRIs [81]. However, due to their varying degrees of receptor affinities, TCAs are associated with a high risk of adverse effects.

#### 3.1.2. Anti-Inflammatory Drugs and Vitamin D

Beyond monoamine-based antidepressants, interventions targeting neuroinflammation and neurotrophic pathways have gained attention. Nonsteroidal anti-inflammatory drugs (NSAIDs) may alleviate PSD symptoms by inhibiting inflammatory pathways [82]. However, their effects may be time-dependent, reducing early-onset depression while potentially increasing late depression risk [83]. Minocycline, a tetracycline antibiotic with anti-inflammatory properties, exhibits particular promise by increasing neuronal viability and reducing infarct volume following cerebral ischemia [84], as well as reversing the pathogenic phagocytic activity of neurotoxic M1 microglia [85], thereby alleviating depressive-like behavior [86]. These findings directly connect to the microglial polarization mechanisms discussed in Section 2.2, suggesting minocycline may exert its antidepressant effects by shifting microglia from pro-inflammatory M1 toward neuroprotective M2 phenotypes. According to previous research, vitamin D deficiency may be a risk factor for depression [87,88]. Gu et al. found a higher prevalence of vitamin D deficiency and insufficiency in patients with acute stroke. Low serum vitamin D levels were associated with the development of PSD [89]. Vitamin D’s relevance to PSD stems from its multiple mechanistic links to the pathways discussed earlier. It affects the synthesis of neurotransmitters, such as serotonin and DA, is involved in changes in brain morphology [90,91], and also stimulates the BDNF signaling pathway and enhances neuroplasticity, directly engaging the neurotrophic mechanisms described in Section 2.4 and potentially contributing to neurological recovery and PSD amelioration [92].

### 3.2. TCM Therapy

TCM has gained increasing recognition in depression treatment, with studies demonstrating efficacy when combined with conventional therapies [93]. TCM interventions target multiple nodes within the PSD network, including neuroinflammation, HPA axis dysfunction, and neurotrophic support, aligning with the integrated pathogenesis model discussed in Section 2.

#### 3.2.1. Herbal Formulations and Extracts

TCM formulas combined with SSRIs have demonstrated safety and efficacy in depression treatment. A systematic review indicated that Chaihu-Shugan-San, in particular, offers beneficial adjunctive effects when combined with SSRIs [94]. Mechanistic studies reveal that Chaihu-Shugan-San regulates microglial polarization through multiple intracellular pathway, inhibiting neuroinflammation and ameliorating PSD symptoms [41,95], which directly engage the microglial mechanisms discussed in Section 2.2. In animal PSD models, researchers found that traditional Chinese medicines can inhibit the activation of the HPA axis, reduce the levels of serum CRH, ACTH and GCs, inhibit the activation of brain glial cells and decrease the expression of inflammatory factors [96,97]. These effects directly counter the HPA hyperactivation described in Section 2.3. Specific herbal extracts have also shown promise. St. John’s wort normalizes the overactive HPA axis through antioxidant and anti-inflammatory properties. Gastrodin, derived from Tianma, enhances intestinal barrier function and reduces pro-inflammatory cytokines in mice [98], with efficacy in alleviating depression-associated behavioral deficits [99].

#### 3.2.2. Acupuncture and Moxibustion

Acupuncture (ACUP) is widely recognized as an effective and safe therapeutic approach for PSD [100,101]. ACUP can exchange information between immune–neurological–endocrine–microbial metabolism through the brain–intestinal axis, further balancing the structure of intestinal flora, maintaining intestinal homeostasis, improving the dysfunction of the HPA axis, and inhibiting inflammatory responses, thus improving the symptoms of PSD patients [102]. A meta-analysis highlighted ACUP as the most effective intervention for improving depressive symptoms after stroke, surpassing other many non-pharmacological therapies [10]. Another review of seven studies confirmed its superior efficacy over control groups [103]. Combination approaches show particular promise. Meta-analyses demonstrate that ACUP plus fluoxetine is superior to fluoxetine alone [101]. In addition, a network meta-analysis of 62 studies further established that compared with Western medicine (WM), ACUP alone or in combination with repetitive transcranial magnetic stimulation (rTMS), TCM, WM, or TCM with WM seemed to be more effective in improving depression symptoms of PSD [100]. Furthermore, moxibustion has also been validated as an effective PSD treatment in multiple clinical trials [104,105].

### 3.3. Psychological Therapy

In recent years, various psychological interventions have been implemented to reduce or eliminate mental health concerns in poststroke patients, such as music support therapy (MST), supportive psychotherapy, self-efficacy training, cognitive–behavioral therapy (CBT), and mindfulness-based interventions. These non-pharmacological approaches complement the multi-system treatment framework by directly targeting the psychological and behavioral dimensions of PSD, while also exerting indirect effects on neurobiological pathways. MST is a new interdisciplinary field that incorporates medicine, psychology, and physics, and has gradually been applied in the clinical treatment of CNS diseases [106]. MST have a stabilizing effect on enhancing depressive symptoms, daily living skills, and serum 5-HT levels in people living with stroke [107,108]. Additionally, CBT has the most extensive evidence base among psychological interventions for PSD. Several studies have demonstrated the effectiveness of CBT and its combination with antidepressants in the treatment of PSD [109,110]. By helping patients identify and modify negative thought patterns and behaviors, CBT facilitates early post-stroke recovery and reduces depressive symptoms. Other psychological interventions offer additional options for patients based on individual preferences and clinical presentation.

### 3.4. Neuroregulation

Neuroregulation is a biomedical engineering technique that uses invasive or non-invasive techniques to alter the transmission of signals in the nervous system, to regulate the activity of neurons and their neural networks, and ultimately cause specific changes in brain function. These techniques can induce both rapid local functional changes and sustained alterations in neuronal plasticity and circuit connectivity. Importantly, neuroregulation targets multiple nodes within the PSD network-including monoaminergic transmission, neuroinflammation, and neuroplasticity-offering a mechanistically grounded approach that aligns with the integrated pathogenesis model discussed in Section 2.

#### 3.4.1. Invasive Neuromodulation Techniques

Deep brain stimulation (DBS) represents the most invasive neuromodulation approach for PSD. Research has found that the long-term effects observed here with chronic DBS resemble the effects of slower-acting antidepressants, particularly SSRIs [111], suggesting convergence on common downstream neuroplastic mechanisms. Vagus nerve stimulation (VNS), initially approved for epilepsy treatment, is increasingly investigated for psychiatric applications including depression, anxiety disorders, and Alzheimer’s disease [112]. Preclinical studies have elucidated multiple neuroprotective mechanisms relevant to PSD: VNS reduces inflammatory injury to neurons, inhibits pyroptotic responses [113], promotes angiogenesis, and enhances axonal plasticity [114]. Clinical studies reveal that VNS therapy can alleviate blood–brain barrier and colonic barrier damage after cerebral ischemia/reperfusion by modulating immune cells, thereby mitigating systemic inflammatory responses [115], which directly engages the neuroinflammatory pathways discussed in Section 2.2. A promising approach in addressing PSD is the use of repetitive auricular vagus nerve stimulation (ta-VNS), a non-invasive treatment that gently stimulates the peripheral auricular branch of the vagus nerve [116]. Double-blind, randomized controlled trials have shown that the synergistic approach of combining ta-VNS with conventional treatment demonstrates remarkable efficacy and tolerability in managing PSD [117].

#### 3.4.2. Non-Invasive Neuromodulation Techniques

Non-invasive brain stimulation techniques [118], including transcranial direct current stimulation (tDCS) [119] and rTMS [120], have been validated by multiple meta-analyses as effective treatments for PSD. The tDCS is an emerging non-pharmacological treatment that applies direct current through scalp electrodes to modulate activity in mood-regulating brain regions. A systematic review of seven randomized controlled trials involving 217 patients by Marchina et al. demonstrated significant reductions in depression scale scores following tDCS sessions [121]. Similarly, Valiengo et al. sampled 48 stroke survivors in a randomized controlled trial and found that those receiving tDCS scored lower on the Hamilton Depression Rating Scale, confirming its therapeutic benefit in PSD [120].

rTMS induces excitability changes in the motor cortex via a magnetic field generated through a scalp coil. Beyond its direct neuromodulatory effects, rTMS has been shown to enhance synthesis and release of 5-HT, DA, and NE, modulate inhibitory and excitatory amino acid neurotransmitters, and mitigate inflammatory responses [122]. As such, rTMS efficacy and safety are increasingly accepted in PSD treatment [123]. However, clinicians must remain vigilant regarding potential risks, as TMS can precipitate dangerous events in stroke patients, including epilepsy [124].

### 3.5. Other Treatments

Beyond pharmacological, psychological, and neuromodulatory interventions, several additional therapeutic approaches have shown promise in PSD management. Notably, home-based exercise has gained popularity in recent years as most stroke patients are discharged home [125]. Moderate physical activity has been shown to have a protective effect against cerebrovascular accidents, such as strokes [126], and may prevent depression onset [127,128]. Mechanistically, continuous progressive training in PSD patients reduces CRH secretion, inhibits excessive glutamate release, and restores HPA axis function while promoting hippocampal neurogenesis. Additionally, mind–body exercises like Tai Chi and Qigong, bridging traditional and complementary medicine, have demonstrated efficacy in enhancing quality of life, motor function, daily living activities, and mitigating depressive symptoms in stroke survivors [36]. Although their conclusions all imply that physical activity has a positive effect on relieving depression in stroke patients [129], related studies have also found that physical activity has no clear impact on long-term depressive symptoms in stroke survivors [130,131]. Electroconvulsive therapy (ECT) has emerged as an effective non-pharmacological treatment for PSD [132], with randomized controlled trials demonstrating its efficacy alongside electroacupuncture [133]. Hyperbaric oxygen treatment (HBOT) is a therapy providing patient with 100% pure oxygen at a pressure above normal atmosphere [134]. Several clinical studies have confirmed the effectiveness and safety of HBOT in PSD [135].

Pharmacological therapy remains the mainstay of PSD treatment in clinical practice, with SSRIs, SNRIs, and TCAs targeting monoaminergic deficits as discussed in Section 2.1. A search of ClinicalTrials.gov reveals that clinical drug studies have already been conducted specifically in PSD populations [136]. Among them, NCT00177424 assessed the effectiveness of sertraline in preventing the occurrence of PSD, while NCT02472613 and NCT01174394 investigated the therapeutic effects of ACUP and fluoxetine, respectively, for PSD. Beyond conventional monoaminergic agents, novel pharmacological targets are under investigation. NCT06759558 is evaluating whether the oral synthetic allopregnanolone analogue zuranolone is safe and effective in alleviating depressive symptoms in PSD patients. NCT05932550 tested the CCR5 antagonist maraviroc in a proof-of-concept trial for PSD, suggesting it may be a tolerable and potentially effective pharmacotherapy [137]. NCT04876066 assessed the feasibility of transmucosal ketamine administration in PSD patients. In addition, NCT03147053 evaluated the efficacy and safety of the traditional Chinese medicine Jiedu Tongluo Granule in the treatment of PSD. However, antidepressants have limitations: long-term use may lead to drug resistance, therapeutic effects are typically delayed by several weeks, and potential risks including bleeding, fractures, and other adverse effects require careful consideration [73,138]. The expanding therapeutic landscape for PSD now encompasses multiple mechanistically distinct approaches that align with the multi-system network model. This diversity of therapeutic options reflects the complex pathogenesis of PSD and supports a personalized, multi-target treatment approach. Future research should prioritize large prospective studies to clarify long-term safety profiles, identify optimal combination strategies, and develop biomarkers that guide treatment selection based on dominant pathogenic mechanisms in individual patients.

## 4. The Underlying Mechanisms of MSCs in the Treatment of PSD

**Figure 3:**
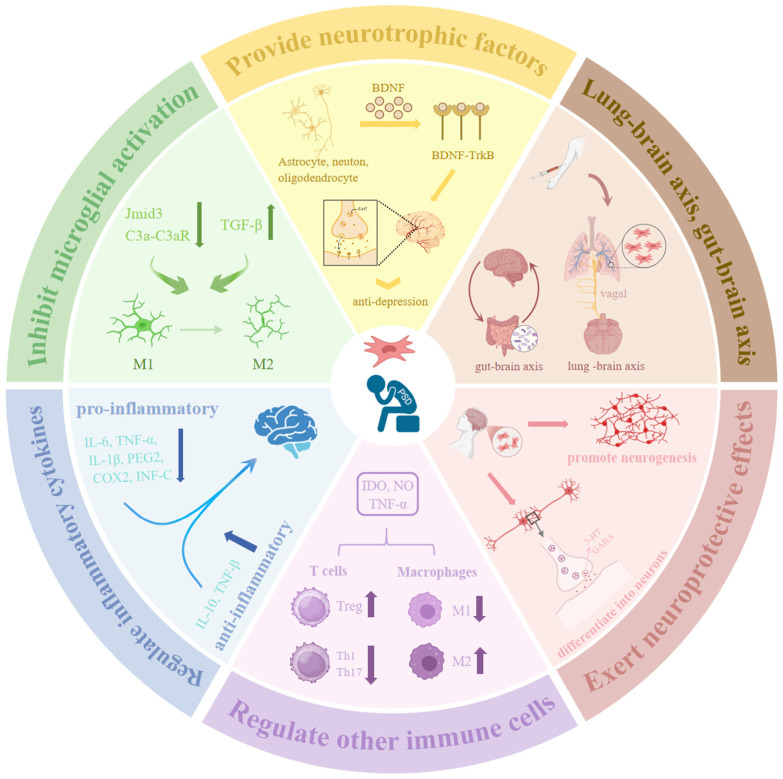
The underlying mechanisms of MSCs in the treatment of PSD mainly include the following: inhibit microglial activation, regulate inflammatory cytokines, regulate other immune cells, exert neuroprotective effects, through the lung–brain axis and gut–brain axis, and provide neurotrophic factors. PSD, post-stroke depression; BDNF, brain-derived neurotrophic factor; TrkB, tropomycin receptor kinase B; 5-HT, 5-hydroxytryptamine; GABA, γ-aminobutyric acid; IDO, indoleamine 2,3-dioxygenase; TNF-α, tumor necrosis factor-α; IL-6, interleukin-6; TNF-α, tumor necrosis factor-α; IL-1β, interleukin-1β; PEG2, prostaglandin E2; COX2, cyclooxygenase 2; INF-C, interferon-C; IL-10, interleukin-10; TNF-β, tumor necrosis factor-β; Jmjd3, jumonji domain-containing protein 3; TGF-β, transforming growth factor-β; MSCs, mesenchymal stem cells.

In recent years, MSCs therapy have emerged as a particularly promising approach, given the critical roles of MSCs in tissue repair, angiogenesis, and immunomodulation, which directly engage the neuroinflammatory and neurotrophic pathways central to PSD pathology. MSCs derived from diverse sources, including umbilical cord, adipose tissue, and bone marrow, have been extensively investigated. Studies demonstrate that human umbilical cord-derived MSCs (hUC-MSCs) preserve blood–brain barrier integrity, mitigate neuroinflammation and neuronal damage, and effectively ameliorate depressive- and anxiety-like behaviors [139,140]. Adipose-derived MSCs (ADSCs) exert anti-inflammatory effects and alleviate chronic mild stress-induced depressive-like behavior [141], with ADSC-derived exosomes (ADSC-Exos) further demonstrating efficacy through inhibition of microglial activation and NLRP3 inflammasome formation [142,143]. Bone marrow-derived MSCs (BM-MSCs) alleviate depressive behavior by promoting neurogenesis [144] and modulate the serotonergic system via the vagus nerve–brain axis [145]. Despite this accumulating evidence, studies specifically investigating MSC therapy for PSD remain limited. Therefore, this section synthesizes current understanding of MSC-based therapeutic mechanisms in depression, alongside the unique biological properties of MSCs to postulate their potential therapeutic role in PSD. We critically examine key mechanistic pathways through which MSCs may ameliorate PSD pathology, specifically including modulation of neuroinflammatory cascades, Exertion of neuroprotective effects, and provision of NTFs (Figure 3). This integrated analysis establishes a translational framework for future therapeutic development targeting the neuropsychiatric sequelae of stroke. Before examining the specific mechanisms of MSC therapy in detail, it is instructive to compare its therapeutic properties with those of conventional PSD treatments summarized in Section 3. Table 2 highlights these key mechanistic distinctions.

### 4.1. Anti-Inflammatory Function

Neuroinflammation is widely recognized as a pivotal factor in the pathogenesis of PSD, with microglial activation representing a central cellular mechanism and pro-inflammatory cytokines serving as key molecular effectors (Section 2.2). MSCs exert potent immunomodulatory effects on CNS inflammation through multilevel mechanisms that collectively reshape the inflammatory microenvironment. The following sections elaborate on three primary and interconnected mechanisms: inhibition of microglial activation, regulation of inflammatory cytokine balance, and modulation of peripheral immune cells that infiltrate or communicate with the CNS.

#### 4.1.1. Inhibit Microglial Activation

Microglia, the resident innate immune cells within the CNS, serve as central orchestrators of neuroinflammatory responses [146]. Neuroinflammation severely affects the morphology and function of microglia, disrupts their proliferation/apoptosis sequence, and impedes their signaling pathways, which in turn damages the entire cerebral nervous system, leading to depression-like behaviors such as memory loss, loss of pleasure, and hyperalgesia [147]. Critically, microglial activation has been consistently observed in PSD patients [5,41], establishing this cellular population as a relevant therapeutic target. Activated microglia exhibit functional polarization into classically activated (M1; pro-inflammatory) and alternatively activated (M2; anti-inflammatory) phenotypes [148]. The M1/M2 balance determines the net inflammatory milieu, with M1 predominance driving neurotoxicity and M2 polarization promoting tissue repair. Experimental evidence demonstrates that MSCs modulate this balance through multifaceted mechanisms. Firstly, direct paracrine signaling promotes M2 polarization. When BV2 cells are co-cultured with MSCs, the TGF-β1 secreted by MSCs promotes the phenotypic polarization of BV2 cells towards M2 microglial cells, thereby exhibiting antidepressant potential. Secondly, specific molecular pathways mediate MSC-induced microglial reprogramming. Nuclear factor E2-related factor 2 (Nrf2) and Toll-like receptor 4 (TLR4) play critical roles in ADSCs-mediated microglial phenotypic regulation, with ADSC treatment shifting microglia toward M2 while suppressing inflammation [146,149,150]. Thirdly, epigenetic regulation contributes to MSC effects. HUC-MSCs facilitate immunoprotective M2 transition by downregulating the pro-inflammatory epigenetic regulator Jumonji domain-containing protein 3 (Jmjd3) [151] and by inhibiting C3a-C3aR complement signaling [152]. Fourthly, MSC-derived exosomes (MSC-Exos) recapitulate these effects. Depression upregulates M1 markers (Iba1, iNOS) while inhibiting M2 characteristics (Arg1, CD206); these pathological changes are reversed within one week of MSC-Exo treatment [153], demonstrating rapid anti-inflammatory effects. Collectively, MSC-induced microglial reprogramming shifts the balance from neurotoxic M1 toward neuroprotective M2 phenotypes, representing a fundamental mechanism through which MSCs exert antidepressant effects.

#### 4.1.2. Regulate Inflammatory Cytokines Balance

Beyond cellular regulation, MSCs directly modulate the molecular mediators of neuroinflammation. The pathogenesis of PSD is closely associated with dysregulation of pro-inflammatory cytokines including TNF-α, IL-1β, IL-6, and iNOS [154], and anti-cytokine therapies have demonstrated efficacy in PSD [82]. MSCs exert potent antidepressant effects by fundamentally reshaping this cytokine balance [25]. MSCs consistently downregulate pro-inflammatory cytokines while upregulating anti-inflammatory mediators. MSC therapy effectively reduces expression of IL-1β, IL-6, TNF-α, prostaglandin E2 (PGE2), and cyclooxygenase-2 (COX2). Concurrently, it potently enhances anti-inflammatory cytokines, with IL-10 being the most consistently reported effector, alongside documented upregulation of tumor necrosis factor-beta (TNF-β). This bidirectional regulatory effect has been extensively validated across diverse depression models. In chronic unpredictable mild stress (CUMS) models, MSC treatment inhibits pro-inflammatory factor expression [146]. In a recent study by Wang (2024) [155], using a rat model of depression induced by CUMS and lipopolysaccharide (LPS) co-stimulation, hUC-MSC therapy, even under persistent inflammatory attack, effectively reduced pro-inflammatory cytokines (e.g., IL-6 and TNF-α) and increased anti-inflammatory cytokines (e.g., IL-10 and TNF-β). This finding is in agreement with the results of another experiment [156]. Studies by Lee (2020) and Li (2024) also consistently reported that MSC transplantation enhances IL-10 expression while suppressing pro-inflammatory factors such as Interferon-gamma (IFN-γ) and TNF-α [14,153]. Paracrine mechanisms mediate these effects, as MSC-derived extracellular vesicles similarly reduce brain IL-1β and TNF-α while alleviating depressive behaviors [157]. In summary, reshaping the inflammatory balance by suppressing pro-inflammatory and enhancing anti-inflammatory factors constitutes a pivotal mechanism through which MSCs from diverse sources exert neuroprotective and antidepressant effects across multiple depression models.

#### 4.1.3. Regulate Other Immune Cells

The immunomodulatory capacity of MSCs extends beyond resident microglia to peripheral immune cells that communicate with the CNS. This regulatory role is critical for the prognosis of neuroinflammation-related diseases. MSCs primarily mediate their immunosuppressive effects on macrophages and T cells through the secretion of soluble factors such as indoleamine 2,3-dioxygenase (IDO), TGF-β, and NO [158]. Macrophages represent a key peripheral target. Macrophages, as the main cells of the peripheral immune system, have been proved to participate in the prognosis of stroke via secreting pro-inflammatory cytokines, such as TNF-α and IL-6, and anti-inflammatory cytokines, such as IL-10 [159]. BM-MSCs themselves, and particularly the EVs derived from tetramethylpyrazine-preconditioned BM-MSCs, drive macrophage polarization towards the anti-inflammatory M2 phenotype, thereby exerting neuroprotective and tissue-repairing effects in ischemic stroke [160]. Hypoxia-preconditioned MSC-small extracellular vesicles (MSC-sEVs) effectively suppress the infiltration of macrophages into the damaged area and the accumulation of local microglia after cerebral ischemia, thereby alleviating neuroinflammation and synergistically promoting neurological functional recovery and vascular remodeling [161]. ADSC-Exos have been shown to alleviate chronic inflammation by reducing the number of iNOS+ M1 macrophages and restoring the population of CD163+ M2 macrophages [162]. T cells are also modulated by MSCs. In pro-inflammatory milieus containing IFN-γ from CD4+ or CD8+ T cells, BMSCs acquire enhanced T cell suppressive properties [163]. BM-MSCs reduce systemic Th1-derived IFN-γ, inhibiting neuronal JAK/STAT1 signaling and downstream chemokine CCL8 expression, thereby blocking microglia-mediated aberrant synaptic elimination and rescuing depressive-like behaviors [164]. hUC-MSCs suppress T lymphocyte proliferation, downregulate the Th17 transcription factor RORγt and Th17 proportion, while increasing regulatory T cell (Treg) proportions [163]. Neutrophils represent an emerging target. Following ischemic stroke, recruited neutrophils release reactive oxygen species, metalloproteinases, and neutrophil extracellular traps, exacerbating blood–brain barrier damage [165,166]. Neutrophils exhibit functional heterogeneity, with N1 phenotypes exacerbating inflammation and N2 phenotypes potentially aiding neural repair [167,168]. MSC-derived supernatant and cell lysate directly act on neutrophils, regulating their polarization from pro-inflammatory N1 toward anti-inflammatory N2 phenotypes, ultimately alleviating neuroinflammation after ischemic brain injury [169].

MSCs exert their anti-inflammatory effects through an integrated, multi-level network that targets both central and peripheral immune compartments. They reprogram resident microglia from M1 toward M2 phenotypes, reshape the cytokine milieu by suppressing pro-inflammatory and enhancing anti-inflammatory mediators, and modulate infiltrating and peripheral immune cells including macrophages (M2 polarization), T cells (Th1/Th17 suppression, Treg promotion), and neutrophils (N1 to N2 shift). This comprehensive immunomodulatory capacity directly engages the neuroinflammatory pathways detailed in Section 2.2 and provides a cellular and molecular basis for the antidepressant effects observed with MSC therapy. However, it should be noted that while these mechanisms are well-established in related disease models including stroke, lupus, and chronic inflammation, direct evidence within PSD-specific models remains relatively limited, representing an important direction for future investigation.

### 4.2. Exert Neuroprotective Effects

Beyond their immunomodulatory functions, MSCs exert direct neuroprotective effects that address core pathological features of PSD, particularly hippocampal impairment and neural circuit dysfunction. These effects operate through two complementary mechanisms: promoting endogenous neurogenesis and differentiating into functional neuronal lineages to directly replenish damaged neural circuits.

#### 4.2.1. Promote Neurogenesis

Impaired neurogenesis in the HIP dentate gyrus (DG) constitutes a core pathological feature of depression, with clinical imaging confirming significant hippocampal atrophy in PSD patients. MSCs directly target this deficit by modulating the neurogenic microenvironment and promoting the generation of new neurons.

Firstly, MSCs enhance hippocampal neurogenesis through paracrine delivery of NTFs. Encapsulated MSCs (eMSCs) continuously release BDNF, vascular endothelial growth factor (VEGF), and fibroblast growth factor 2 (FGF2), activating neurogenic pathways in the hippocampal subventricular zone and DG, thereby reversing depressive behaviors [170]. Studies using hUC-MSCs to treat depressive rats demonstrate that both MSCs and their extracellular vesicles enhance dendritic complexity and increase doublecortin X (DCX)+ cell density in the subgranular zone (SGZ) [157], consistent with multiple reports confirming MSC capacity to boost HIP neurogenesis [171]. Secondly, MSCs create a microenvironment conducive to neuronal survival and synaptic remodeling. BM-MSCs suppress inflammation and oxidative stress in the hippocampus, thereby restoring plasticity in emotion-related neural circuits [172]. Given that the hippocampus serves as a core hub within the emotional brain network, this synaptic restoration directly contributes to behavioral improvement. Thirdly, MSCs exert anti-apoptotic effects that preserve existing neurons. HUC-MSCs reduce the number of TUNEL+ and cleaved caspase-3/NeuN+ cells in brain regions of CUMS models, lower the Bax/Bcl-2 ratio, and inhibit hippocampal neuronal apoptosis [139,143]. This neuroprotective effect complements the pro-neurogenic actions to maintain hippocampal integrity. Fourthly, MSCs restore normal neuronal activity patterns across distributed brain networks. By restoring normal c-Fos expression patterns, MSCs correct aberrant neuronal activity across multiple brain regions and reconstruct functional neural networks [139].

In summary, MSCs target the core pathology of hippocampal neurogenesis impairment through multi-dimensional regulation: they promote neural progenitor proliferation, enhance neuronal survival, prevent apoptosis, and restore normal circuit activity. This integrated approach to neural repair establishes a crucial interventional strategy for PSD treatment that directly engages the neurotrophic mechanisms discussed in Section 2.4.

#### 4.2.2. Differentiate into Neurons

Beyond promoting endogenous repair, MSCs possess trans-germ layer differentiation potential, enabling their directed differentiation into specific neuronal subtypes to directly replenish neural circuits. MSCs can differentiate into specific neuronal lineages relevant to mood regulation. Under appropriate induction conditions, stem cells differentiate into central serotonin-like neuronal cells capable of releasing 5-HT [173]. Additionally, MSCs can be induced to differentiate into GABAergic interneurons, which consistently increase GABA release upon implantation [174,175]. MSCs also differentiate into Schwann cell-like phenotypes, participating in peripheral nerve myelination and regeneration [93,176,177], though this mechanism is more relevant to peripheral nerve injury than central mood regulation. Critically, MSC-derived neurons integrate functionally into host neural circuits. Differentiated neurons not only adopt neuronal morphologies but also establish synaptic connections with existing networks. They modulate emotional behavior through multiple routes: by directly stimulating serotonergic neurons in the dorsal raphe nucleus (DRN) [145], by releasing monoaminergic neurotransmitters including 5-HT and GABA [143,178], and by integrating into and modulating the activity of mood-regulating circuits.

MSCs exert their neuroprotective effects through integrated, complementary mechanisms. They promote endogenous neurogenesis by delivering NTFs, suppressing apoptosis, and creating a supportive microenvironment for neuronal survival and synaptic plasticity. Concurrently, they possess the capacity to differentiate into functional neurons that integrate into host circuits and directly modulate neurotransmitter release. This multi-pronged approach directly targets the neurotrophic deficits and hippocampal impairment detailed in Section 2.4, providing a cellular foundation for the sustained antidepressant effects observed with MSC therapy.

### 4.3. Regulate the Peripheral–Central Communication Axes

Beyond their direct effects on CNS resident cells, MSCs modulate peripheral–central communication pathways that profoundly influence brain function. Two axes have emerged as particularly relevant to PSD: the lung–brain axis, through which intravenously infused MSCs may transmit neural signals to mood-regulating centers, and the GBA, through which MSCs restore microbial homeostasis and reduce systemic inflammation. These peripheral mechanisms expand the therapeutic reach of MSCs beyond the blood–brain barrier and provide additional routes for modulating the neuroinflammatory and neuroendocrine pathways discussed in Section 2.

#### 4.3.1. Lung–Brain Axis

A growing body of evidence suggests that MSCs may exert central effects through direct interaction with peripheral sensory neurons, particularly those innervating the lungs. This paradigm shifts the traditional view that intravenously infused MSCs must cross the blood–brain barrier to exert antidepressant effects. The anatomical basis for this mechanism is well-established: following intravenous infusion, the majority of MSCs become transiently lodged in the pulmonary microvasculature [178]. The lungs are densely innervated by the vagus nerve, which serves as a bidirectional communication highway between peripheral organs and the CNS. This anatomical arrangement positions MSCs to interact with lung-innervating sensory neurons and transmit signals to the brain without requiring CNS penetration. Direct evidence supports MSC-neuron signaling. Huang et al. demonstrated significant neural activation within the DRN serotonergic system following MSC injection [145]. In vivo electrophysiological and patch-clamp recordings confirmed that intravenous MSC infusion directly activates DRN serotonergic neurons. This neural activation occurs rapidly, consistent with a neurally mediated rather than blood-borne mechanism. The paracrine nature of this interaction is increasingly understood. Recent reports demonstrate that MSCs improve osteoarthritis pain by directly modulating joint-innervating sensory neurons through paracrine effects, rather than through anti-inflammatory or cartilage repair activities [179,180]. This establishes precedent for direct MSC-sensory neuron communication. In the pulmonary context, MSC-derived prostaglandin E2 (PGE2) may modulate acetylcholine release from pulmonary parasympathetic nerves [181], potentially altering vagal afferent signaling to the brain. Additional pathways may contribute. The pulmonary microbiome could respond to MSC-induced microenvironmental changes and thereby deliver different information to the CNS [182]. Profiling MSC-derived bioactive molecules may enable targeted modulation of the lung–brain axis for psychiatric interventions.

#### 4.3.2. Gut–Brain Axis

GBA, a bidirectional communication network between the gastrointestinal tract, its microbial inhabitants, and the CNS, has emerged as a critical mediator of mood disorders [3,183]. Stroke disrupts intestinal integrity and alters gut microbiota, leading to increased intestinal permeability, bacterial translocation into the circulation, systemic inflammation, and heightened risk of post-stroke complications including PSD [184]. Clinical evidence firmly establishes GBA involvement in PSD. Compared to healthy controls, PSD patients exhibit significantly altered gut microbial composition, including increased abundance of Bacteroidetes, Proteobacteria, and Fusobacteria and reduced Firmicutes [185]. Pathogenic Enterobacteria increase while probiotics decrease [184,186,187]. Critically, patients with post-stroke comorbid cognitive impairment and depression show reduced levels of short-chain fatty acid (SCFA)-producing bacteria including Fusicatenibacter and Lachnospiraceae [188]. Since SCFAs exert anti-inflammatory and neuroprotective effects, their depletion may directly contribute to PSD pathogenesis. MSCs restore microbial homeostasis through multiple mechanisms. Metagenomic analysis reveals that MSC treatment ameliorates dysbiosis of specific bacterial genera, including Bacteroides acidifaciens, Lactobacillus gasseri, L. reuteri, and L. intestinalis, optimizing microbial composition and abundance [189]. Fecal microbiota transplantation (FMT) experiments confirm that microbiota from MSC-treated mice, when transferred to recipient animals, specifically regulate gut microbial communities—decreasing Bacteroides, Christensenella, Roseburia, and Coprococcus while increasing Xenorhabdus, Sutterella, and Acinetobacter [190].

MSCs engage two complementary peripheral–central communication axes that extend their therapeutic reach beyond direct CNS actions. The lung–brain axis enables rapid neural signaling from peripherally lodged MSCs to mood-regulating centers including the DRN, directly modulating serotonergic activity. The GBA enables sustained restoration of microbial homeostasis, reducing systemic inflammation and creating a peripheral environment conducive to CNS recovery. Together, these peripheral mechanisms provide additional routes through which MSCs address the multi-system nature of PSD pathology, complementing their direct anti-inflammatory and neuroprotective effects within the CNS.

### 4.4. Provide Neurotrophic Factors

Accumulating evidence indicates that transplanted MSCs mediate therapeutic benefits primarily through secretion of NTFs rather than solely through graft survival and host cellular integration [191]. This paracrine mechanism directly engages the neurotrophic deficits central to PSD pathogenesis (Section 2.4) and provides a sustained, multi-factor intervention that supports neuronal health, plasticity, and survival.

#### 4.4.1. BDNF as a Core Mediator of MSC Therapeutic Effects

BDNF represents the most extensively studied NTFs in depression research. Anti-depressant treatments consistently induce BDNF mRNA expression in neurons [192], astrocytes [193], and microglia [193], and BDNF upregulation may be necessary for antidepressant response [67]. MSCs leverage this critical pathway through multiple mechanisms. Firstly, MSCs directly enhance BDNF expression in host brain tissue. A pioneering study demonstrated that ADSCs differentiated into glial-like stem cells expressing functional glutamate transporters were transplanted into Flinder Sensitive Line rats (FSLs), a genetic depression model [194]. After 31 days, MSC transplantation produced long-term attenuation of depressive-like behaviors including reduced motivation, impaired novelty exploration, and anhedonia. This behavioral improvement was mediated by stimulation of BDNF increases in host brain tissue. Secondly, MSC therapy activates the BDNF-TrkB signaling cascade. MSCs upregulate both BDNF and its high-affinity receptor TrkB. Activation of the BDNF-TrkB pathway modulates neuroinflammation and preserves hippocampal integrity [195,196], directly countering the hippocampal atrophy and inflammatory burden characteristic of PSD. Thirdly, BDNF-TrkB signaling links to monoaminergic regulation. MSCs injected into the nucleus of the solitary tract induce BDNF-TrkB-dependent release and activation of 5-HT in the DRN, exerting anti-anxiety and anti-depression effects [145]. This provides a direct mechanistic bridge between neurotrophic support and the monoaminergic deficits discussed in Section 2.1.

#### 4.4.2. Multi-Factor Neurotrophic Support Beyond BDNF

While BDNF plays a central role, MSCs secrete a broad spectrum of NTFs that synergistically support neuronal function and plasticity. Nerve growth factor (NGF) represents another critical NTF frequently diminished in the CNS of depression patients. MSC-treated mice exhibit mild BDNF increases alongside significant NGF gene expression elevation [155], suggesting coordinated upregulation of multiple neurotrophic systems. MSCs secrete additional NTFs relevant to neurogenesis and neuronal survival, including GDNF, ciliary neurotrophic factor (CNTF), FGF2, and VEGF [197]. MSC transplantation demonstrably upregulates expression levels of VEGF, CNTF, and their cognate receptors [198]. Intrinsic changes in VEGF and CNTF pathways independently contribute to amelioration of depression-like behavior. The functional consequence of this multi-factor support is enhanced neurogenesis. MSCs transplanted into FSL rats significantly increased neurogenesis in hippocampal and DG regions, correlating with improved depression-like behaviors [144]. This neurogenic effect directly addresses the hippocampal impairment central to PSD pathology.

Collectively, MSCs offers a method for continuous supply of a cocktail of exogenous NTFs and upregulating intrinsic expression of NTFs and their receptors. This synergistic action fosters neurogenesis, establishing a novel therapeutic strategy for PSD.

## 5. Conclusions, Final Remarks and Future Perspectives

PSD arises from the complex interplay of multiple interdependent systems rather than a single pathological mechanism. Monoaminergic dysfunction constitutes the direct neurochemical substrate of depressive symptoms, while neuroinflammation, HPA axis dysregulation, and neurotrophic imbalance function as interconnected amplifying systems that perpetuate and exacerbate the condition. Current pharmacological treatments target monoaminergic deficits but face significant limitations including delayed onset, drug resistance, and adverse effects. These constraints underscore the urgent need for novel therapeutic strategies that address the multi-system nature of PSD pathology.

MSC therapy has emerged as a particularly promising approach, offering multifaceted therapeutic effects that directly engage the interconnected pathogenic nodes identified in this review. Firstly, they modulate microglial activation (conversion from M1 to M2) by downregulating Jmjd3, inhibiting C3a-C3aR signaling, and upregulating TGF-β. Secondly, MSCs exert a bidirectional regulatory effect of inhibiting pro-inflammatory factors and enhancing anti-inflammatory factors to reshape the inflammatory balance, thereby improving the neuroinflammatory environment and ultimately alleviating depressive-like behavior. Thirdly, this immunomodulatory effect is extended through the secretion of soluble factors like IDO, TGF-β, and NO, which suppress inflammatory macrophages (iM1), Th1, and Th17 cells while restoring anti-inflammatory M2 macrophages and Treg cells, thus mitigating chronic inflammation. Fourthly, beyond immunomodulation, MSCs can promote neurogenesis and induce differentiation into 5-HT and GABA neurons. Fifthly, they also leverage functional connections along the lung–brain and gut–brain axes for targeted systemic regulation. Finally, MSCs provide a sustained source of exogenous NTFs while concurrently upregulating the expression of endogenous NTFs and their receptors within the host. Collectively, these actions contribute to alleviating depressive-like behaviors associated with PSD.

It is important to acknowledge that the mechanistic evidence synthesized here originates predominantly from studies conducted in general depression models rather than from models that specifically recapitulate PSD. This creates a two-step translational gap that must be critically examined. Before the findings from generic depression models can be considered applicable to PSD, they must be validated in models that combine ischemic brain injury with chronic stress, confirming that the therapeutic effects of MSCs persist within a post-stroke neuroinflammatory milieu. Furthermore, even after PSD-specific preclinical evidence is accumulated, it must still be bridged to clinical populations through carefully designed trials that enroll PSD patients and assess both neurological and psychiatric endpoints. A systematic research effort that moves beyond extrapolation is therefore essential. This effort should begin with the establishment of PSD-relevant animal models and the benchmarking of MSC therapy against standard antidepressants in those models, and extend to the optimization of cell delivery parameters tailored to the evolving inflammatory environment of the post-stroke brain. By explicitly recognizing these translational steps, the field can progress from promising but indirect evidence toward rigorous, PSD-focused therapeutic development.

For clinical purposes and basic science experiments, MSCs can be isolated from different tissues, in which bone marrow, umbilical cord, and adipose tissues are the most common sources. MSCs isolated from different tissues share core therapeutic mechanisms while exhibiting source-specific advantages and limitations. BM-MSCs represent the gold-standard source for clinical use due to their immunosuppressive properties, low immunogenicity, differentiation capacity, and homing potential, despite the painful and invasive harvesting procedure. They effectively promote hippocampal neurogenesis and modulate serotonergic pathways via the vagus nerve–brain axis. ADSCs offer abundant accessibility as surgical byproducts, long-term culture stability, high exosome yield with superior immune regulatory capabilities, and efficacy in exosome-mediated delivery (e.g., intranasal exosomes suppressing NLRP3 inflammasome and enhancing AMPK/mTOR-autophagy). However, donor variability (age, BMI) and concerns regarding tumorigenicity and low post-transplant survival rates limit their application. HUC-MSCs provide non-invasive isolation, high cell yield, low immunogenicity, high proliferative potential, and cryopreservation feasibility, with unique capacity to preserve blood–brain barrier integrity and exert potent anti-neuroinflammatory effects. However, their therapeutic impact may diminish during later stroke phases due to tissue liquefaction and cyst formation in the subacute and sequelae stages.

The clinical translation of MSC-based therapies faces substantial challenges, and their application in PSD is no exception. A considerable challenge pertains to the reduced survival rate of these cells in the impaired, ischemic state of the brain. Strategies to address this limitation include hypoxic preconditioning, pharmacological preconditioning with compounds such as valproate and lithium, and cytokine mixture pretreatment to enhance MSC resilience and therapeutic potency. Additionally, standardization deficits impede clinical progress. MSCs derived from different sources, passage numbers, and culture conditions exhibit variable secretory profiles and therapeutic efficacy. The adoption of standardized protocols for cell isolation, expansion, characterization, and delivery is essential for consistent clinical outcomes. Future trials must prioritize randomization, blinding, and rigorous controls to minimize bias and ensure validity. Significantly, safety concerns require vigilant monitoring. While phase I trials generally support MSC safety, long-term risks including tumorigenicity and immune rejection necessitate extended follow-up. Autologous MSCs face fewer regulatory restrictions, but donor-derived or genetically modified cells must undergo stringent FDA and EMA approval processes. Future breakthroughs are likely to depend on developing next-generation ‘smart’ MSC-based therapeutics that are scalable, traceable, and regulatable. The integration of CRISPR/Cas9 gene editing with stem cell therapy could enable engineering of MSCs with enhanced survival, targeted homing, and regulated therapeutic protein production.

While recent reviews have valuably surveyed the landscape of stem cell therapy for depression or neurological disorders, they leave important territory uncharted. Leszek et al. (2025) provided a timely overview of stem cell types for major depressive disorder and highlighted the potential of extracellular vesicles and intranasal delivery [24]. Li et al. (2024) offered a comprehensive mechanistic account of stem cell therapy for major depressive disorder, covering neurogenesis, neurotrophic signaling, neuroinflammation, neurotransmitter systems, and HPA axis regulation [25]. Bani Issa et al. (2025) consolidated evidence on MSCs across multiple neurological diseases with an emphasis on shared translational challenges [23]. Yet none of these works addresses the distinct neuropsychiatric context of PSD. The present review advances beyond these foundations by concentrating specifically on MSC therapy for PSD, a setting in which ischemic injury, sterile inflammation, and secondary neurodegeneration converge with mood dysregulation. This disease-specific lens allows the construction of a multi-system network model in which monoamine deficits, neuroinflammation, HPA axis dysfunction, and neurotrophic imbalance are treated not as separate pathways but as interconnected pathogenic nodes that the pleiotropic properties of MSCs are uniquely suited to address. Furthermore, the review articulates a PSD-focused translational agenda that identifies the two-step gap from generic depression models to PSD-specific evidence and from preclinical efficacy to clinical validation, while also giving dedicated attention to mitochondrial transfer, preconditioning strategies, and gene-edited intelligent MSs. By narrowing both the disease and the cell type, the manuscript offers a level of mechanistic integration and translational specificity that complements and extends the contributions of existing broader reviews.

This review synthesizes current understanding of PSD pathogenesis as a multi-system network disorder and evaluates the therapeutic potential of MSCs within this framework. MSCs address the interconnected pathogenic nodes through integrated immunomodulatory, neuroprotective, paracrine, and peripheral–axial mechanisms, including neuroinflammation, monoamine deficits, HPA axis dysfunction, and neurotrophic imbalance. While these findings support the preclinical promise of MSC-based strategies, their translation into PSD therapies must first bridge two critical gaps: from generic depression models to PSD-specific validation, and from preclinical efficacy to rigorous clinical demonstration. The path forward therefore requires systematic optimization in disease-relevant models, standardized protocols for cell production and delivery, and well-designed randomized controlled trials that enroll PSD patients and assess both neurological and psychiatric outcomes. Only by addressing these translational steps can the field move toward safe, effective, and accessible MSC-based therapies for PSD.

## Figures and Tables

**Table 1:** Current therapeutic approaches for PSD and their mechanisms of action.

Treatment Category	Specific Therapy	Proposed Mechanism of Action in PSD	Related Pathogenesis in Section 2
Drug therapy	SSRIs	Block 5-HT reuptake, increasing synaptic 5-HT concentration; alleviate depressive, anxiety, and cognitive symptoms; may prevent PSD development	Section 2.1 Monoamine deficits
	SNRIs	Block reuptake of both 5-HT and NE, increasing synaptic concentrations of both neurotransmitters; activate descending inhibitory pathways	Section 2.1 Monoamine deficits
	TCAs	Block reuptake of 5-HT and NE; efficacy comparable to SSRIs/SNRIs, but with a higher risk of adverse effects	Section 2.1 Monoamine deficits
	NSAIDs	Inhibit cyclooxygenase-mediated inflammatory pathways; effects may be time-dependent, reducing early depression but potentially increasing late-onset risk	Section 2.2 Neuroinflammation
	Minocycline	Shifts microglia from pro-inflammatory M1 to neuroprotective M2 phenotype; improves neuronal viability; reduces infarct volume; reverses pathogenic phagocytic activity	Section 2.2 Neuroinflammation
	Vitamin D	Influences 5-HT and DA synthesis; stimulates BDNF-TrkB signaling and neuroplasticity; deficiency linked to higher PSD risk	Section 2.1 and Section 2.4
TCM therapy	Herbal formulations and extracts	Suppress HPA axis activation, reduce serum CRH, ACTH, GC; inhibit glial activation and inflammatory cytokine expression	Section 2.2 and Section 2.3
	Acupuncture and Moxibustion	Promotes immune–neuro-endocrine–microbial communication via the brain–gut axis; rebalances intestinal flora; ameliorates HPA axis dysfunction; inhibits inflammatory responses	Section 2.1, Section 2.2 and Section 2.3
Psychological therapy	MST, CBT	Elevates serum 5-HT levels, stabilizes mood, and improves daily living skills; often combined with antidepressants to promote early recovery	Section 2.1 Monoamine deficits
Neuroregulation	DBS	Chronic stimulation produces slow-onset antidepressant effects similar to SSRIs, possibly via downstream neuroplasticity mechanisms	Section 2.1 and Section 2.4
	VNS, ta-VNS	Reduces neuronal inflammatory injury and pyroptosis; promotes angiogenesis and axonal plasticity; protects blood–brain barrier and intestinal barrier via immunomodulation	Section 2.2 Neuroinflammation
	tDCS,	Modulates prefrontal and emotion-related cortical activity; reduces depression scores	
	rTMS	Induces excitability changes in the motor cortex; enhances synthesis and release of 5-HT, DA, and NE; regulates excitatory/inhibitory amino acid balance; attenuates neuroinflammation	Section 2.1 and Section 2.4
Other treatments	Physical exercise	Reduces CRH secretion, inhibits excessive glutamate release, restores HPA axis negative feedback; promotes hippocampal neurogenesis	Section 2.1, Section 2.3 and Section 2.4
	ECT	Modulates neurotransmitter systems	Section 2.1
	HBOT	Attenuates neuroinflammation; inhibits serotonin uptake, contributing to antidepressant effects	Section 2.1 and Section 2.2

Note: PSD, post-stroke depression; SSRIs, serotonin-reuptake inhibitors; 5-HT, 5-hydroxytryptamine; SNRIs, serotonin–norepinephrine reuptake inhibitors; NE, norepinephrine; TCAs, tricyclic antidepressants; NSAIDs, Nonsteroidal anti-inflammatory drugs; DA, dopamine; BDNF, brain-derived neurotrophic factor; TrkB, tropomycin receptor kinase B; TCM, traditional Chinese medicine; HPA, hypothalamic–pituitary–adrenal; CRH, corticotropin-releasing hormone; ACTH, adrenal cortical hormone; GC, glucocorticoid; MST, music support therapy; CBT, cognitive–behavioral therapy; DBS, deep brain stimulation; ta-VNS, repetitive auricular vagus nerve stimulation; tDCS, transcranial direct current stimulation; rTMS, repetitive transcranial magnetic stimulation; ECT, electroconvulsive therapy; HBOT, hyperbaric oxygen treatment.

**Table 2:** Comparison of conventional PSD therapies and MSC-based therapy: mechanistic distinctions.

Therapeutic Dimension	Conventional Therapies in Section 3	MSC-Based Therapy in Section 4	Key Distinction
Anti-inflammatory approach	Minocycline shifts microglia from M1 to M2; NSAIDs inhibit cyclooxygenase pathways Inhibits microglial M1 polarization and promotes M2	Downregulates IL-1β, IL-6, TNF-α, PGE2, COX2 and upregulates IL-10, TNF-β; modulates peripheral macrophages, T cells, and neutrophils	Single-target vs. comprehensive immunomodulation
Neurotrophic support	Vitamin D stimulates BDNF signaling	Directly secretes multiple NTFs; activates BDNF-TrkB signaling pathway	Indirect/single-factor vs. multi-factor paracrine delivery
Neurogenesis promotion	Physical exercise promotes hippocampal neurogenesis	Enhances hippocampal neurogenesis via paracrine NTFs; increases DCX+ cell density; restores dendritic complexity; inhibits hippocampal neuronal apoptosis	Activity-dependent induction vs. direct trophic stimulation
Neurotransmitter modulation	SSRIs/SNRIs/TCAs block 5-HT and/or NE reuptake; rTMS enhances monoamine synthesis	Activates DRN serotonergic neurons via the lung–brain vagal axis; differentiates into 5-HT and GABA-releasing neurons; BDNF-TrkB-dependent 5-HT release	Pharmacological blockade vs. neural circuit activation
Peripheral– central communication	Acupuncture engages the gut–brain axis; ta-VNS stimulates vagal afferents	Lung–brain axis: pulmonary-entrapped MSCs modulate pulmonary parasympathetic acetylcholine release via PGE2, potentially altering vagal afferent signaling; gut–brain axis: restores gut microbial homeostasis, modulating specific bacterial genera	Stimulation- dependent vs. microenvironment remodeling
Neural circuit repair	rTMS and tDCS modulate cortical excitability; DBS provides chronic stimulation	Promotes endogenous neurogenesis and synaptic remodeling; differentiates into functional neurons that integrate into host circuits; restores normal c-Fos activity patterns	Neuromodulation vs. structural repair and integration
Coverage of PSD network nodes	Each therapy mainly targets 1–2 nodes	Simultaneously acts on neuroinflammation, neurotrophic deficits, neurotransmitter imbalance, and peripheral–central axes	Narrow vs. multi-node network engagement

Note: PSD, post-stroke depression; MSCs, mesenchymal stem cells; NSAIDs, Nonsteroidal anti-inflammatory drugs; IL-1β, interleukin-1β; IL-6, interleukin-6; TNF-α, tumor necrosis factor-α; COX2, cyclooxygenase 2; IL-10, interleukin-10; TNF-β, tumor necrosis factor-β; BDNF, brain-derived neurotrophic factor; NTFs, neurotrophic factors; TrkB, tropomycin receptor kinase B; DCX, doublecortin X; SSRIs, serotonin-reuptake inhibitors; SNRIs, serotonin–norepinephrine reuptake inhibitors; TCAs, tricyclic antidepressants; 5-HT, 5-hydroxytryptamine; NE, norepinephrine; rTMS, repetitive transcranial magnetic stimulation; DRN, dorsal raphe nucleus; GABA, γ-aminobutyric acid; ta-VNS, repetitive auricular vagus nerve stimulation; tDCS, transcranial direct current stimulation; DBS, deep brain stimulation.

## Data Availability

No new data were created or analyzed in this study. Data sharing is not applicable to this article.

## Abbreviations

The following abbreviations are used in this manuscript:

PSD	post-stroke depression
iPSCs	induced pluripotent stem cells
MSCs	mesenchymal stem cells
CNS	central nervous system
PD	Parkinson’s disease
TBI	traumatic brain injury
NTFs	neurotrophic factors
ACTH	adrenal cortical hormone
BDNF	brain-derived neurotrophic factor
CRH	corticotropin-releasing hormone
DA	dopamine
GDNF	glial cell line-derived neurotrophic factor
GLU	glutamate
IGF-1	insulin-like growth factor-1
IL-1β	interleukin-1β
IL-6	interleukin-6
IL-10	interleukin-10
NE	norepinephrine
TNF-α	tumor necrosis factor-α
5-HT	5-hydroxytryptamine
Tyr	tyrosine
BH4	tetrahydrobiopterin
HPA	hypothalamic–pituitary–adrenal
GC	glucocorticoid
NMDA	N-methyl-D-aspartate
mBDNF	mature BDNF
IDO	indoleamine 2,3-dioxygenase
iNOS	inducible nitric oxide synthase
CCL2	C-C motif chemokine ligand 2
TGF-β	transforming growth factor-β
DST	dexamethasone suppression test
5-HTTLPR	serotonin transporter gene-linked promoter region
TrkB	tropomycin receptor kinase B
TCM	traditional Chinese medicine
DBS	deep brain stimulation
ECT	electroconvulsive therapy
HBOT	Hyperbaric oxygen treatment
MST	music support therapy
CBT	cognitive–behavioral therapy
SNRIs	serotonin–norepinephrine reuptake inhibitors
SSRIs	serotonin-reuptake inhibitors
TCAs	tricyclic antidepressants
tDCS	transcranial direct current stimulation
TMS	transcranial magnetic stimulation
VNS	vagus nerve stimulation
NSAIDs	nonsteroidal anti-inflammatory drugs
ACUP	Acupuncture
WM	Western medicine
rTMS	repetitive transcranial magnetic stimulation
ta-VNS	repetitive auricular vagus nerve stimulation
hUC-MSCs	human umbilical cord-derived MSCs
ADSCs	adipose-derived MSCs
ADSC-Exos	ADSC-derived exosomes
BM-MSCs	bone marrow-derived MSCs
COX2	cyclooxygenase 2
INF-C	interferon-C
Jmjd3	jumonji domain-containing protein 3
PEG2	prostaglandin E2
TNF-β	tumor necrosis factor-β
Nrf2	Nuclear factor E2-related factor 2
GABA	γ-aminobutyric acid
TLR4	Toll-like receptor 4
CUMS	chronic unpredictable mild stress
LPS	lipopolysaccharide
IFN-γ	Interferon-gamma
MSC-sEVs	MSC-small extracellular vesicles
DG	dentate gyrus
DCX	doublecortin X
SGZ	subgranular zone
eMSCs	Encapsulated MSCs
VEGF	vascular endothelial growth factor
FGF2	fibroblast growth factor 2
HIP	hippocampus
DRN	dorsal raphe nucleus
PGE2	prostaglandin E2
GBA	Gut–brain axis
SCFA	short-chain fatty acid
FMT	fecal microbiota transplantation
FSLs	Flinder Sensitive Line rats
NGF	nerve growth factor
CNTF	ciliary neurotrophic factor
